# Fertilization is more effective in alleviating yield loss of waterlogged crops than exogenous growth regulators: a meta-analysis

**DOI:** 10.3389/fpls.2026.1779872

**Published:** 2026-03-11

**Authors:** Hui Xu, Dou-Dou Jin, Ya-Wei Wang, Guo-Hao Liu, Tai-Yu Zhao, Chao Liu, Li-Xin Tian

**Affiliations:** 1 Wuhu Vocational Technical University, College of Landscape and Horticulture, Wuhu, Anhui, China; 2 College of Agronomy/National Engineering Laboratory of Crop Stress Resistance Breeding, Anhui Agricultural University, Hefei, Anhui, China; 3 Huabei Field Comprehensive Scientific Observation Station of the Ministry of Agriculture and Rural Affairs, Anhui Agricultural University, Hefei, Anhui, China; 4 Anhui Liufeng Seed Industry Technology Co., Ltd, Hefei, Anhui, China

**Keywords:** crop stage, crop type, crop yield, management regimes, soil waterlogging

## Abstract

Soil waterlogging ranks among the crucial abiotic stresses that have an impact on crop production and food security. However, the comprehensive assessment of the impact of management practices on crop yield under waterlogging is rarely reported. Thus, we conducted a meta-analysis to investigate the how management practices influence crop yield under waterlogging stress. The results showed that fertilization significantly increased the accumulation of aboveground and root biomass, whereas had no influence on 1000-grain weight. Under waterlogging treatment, fertilization significantly increased the yield of field and potted crops by 123.32% and 36.20%, respectively, and the increase in yield was significantly higher than that of exogenous application of growth regulators. When waterlogging event is carried out during the reproductive growth stage, the increase in crop yield induced by fertilization was higher than that of growth regulator treatment. The increase in wheat yield by regulator treatment is higher than that by fertilization, but the effect on cotton yield was not significant. Overall, this meta-analysis emphasized the potential of fertilization measures in mitigating the harmful effects of waterlogging, providing theoretical basis and technical support for agricultural production.

## 1 Introduction

In recent years, under the combined influence of the continuous deterioration of the global climate environment and the monsoon climate, heavy rainfall has become more frequent and more destructive (Zhou et al., 2025). The Sixth Assessment Report of the Intergovernmental Panel on Climate Change (IPCC) indicated that the risk level of extreme weather events such as flooding events is expected to sharply increase from a global temperature rise of 1.5 °C to 4 °C (https://www.ipcc.ch/report/sixth-assessment-report-cycle/). Meanwhile, the saturated soil conditions caused by compacted soil, low-lying terrain, poor drainage, or high groundwater level severely restricted oxygen to plant roots, and this hypoxia disrupted essential physiological processes, resulting in stunted growth, yield loss, and poorer grain quality. Our previous meta-analysis has identified that waterlogging stress significantly reduced crop yield by 32.9%, which has become one of the important limiting factors affecting China’s food security (Tian et al., 2021a).

Currently, comprehensive researches have thoroughly documented the adverse effects of soil waterlogging on common field crops, mainly including nutrient absorption obstruction, photosynthetic efficiency decline caused under waterlogging conditions, which ultimately seriously lead to yield losses, namely, the average yield reductions of approximately 51.9% for maize, 31.6% for wheat, 42.1% for cotton, and severe losses potentially up to 83% for soybean (Behera et al., 2025; Gangana Gowdra et al., 2025; Qiu et al., 2025; Tian et al., 2021a; Zhang et al., 2025). Meanwhile, multiple studies have also affirmed that the impact of waterlogging on crop yield varies across different growth stages, namely, maize exhibited the highest sensitivity to waterlogging during the seedling stage (Ren et al., 2023; Tian et al., 2020), whereas waterlogging had no significant effect on the yield of durum wheat during the three-leaf stage (Araki et al., 2012). There is a general consensus that as the duration of waterlogging stress prolonged, the reduction in crop yield becomes more pronounced (Huang et al., 2022; Senthamil et al., 2025; Tian et al., 2019a). More importantly, genetic background also plays a crucial role, as materials with different genetic compositions respond differently to waterlogging stress (Pais et al., 2024; Tian et al., 2019b; Xu et al., 2025). Overall, the disparities in the impact of waterlogging on crop yield can be attributed to the crop genotype’s sensitivity (Chen et al., 2024), development stage and duration of waterlogging occurrence (Huang et al., 2022). Nevertheless, for crop yield differences induced by waterlogging stress, implementing tailored agronomic management practices to mitigate its yield-reducing adverse effects is urgently essential.

The influence of waterlogging events on crop yields was obviously affected by soil characteristics, climate conditions, and management regimes (Fan et al., 2024). Various strategies can be implemented to mitigate its adverse effects, including adjusting sowing dates (Li et al., 2022), improving soil drainage systems (Manik et al., 2019), applying growth regulators (Huang et al., 2024; Shao et al., 2025), optimizing fertilization practices (Qi and Wu, 2023; Tian et al., 2021b; Wang et al., 2026), and cultivating waterlogging-tolerant crop varieties (Olorunwa et al., 2022). These aforementioned strategies have been verified to reduce waterlogging-induced yield losses in specific crops, localized environments, or controlled experimental conditions. However, it is still unclear which farmland management regimes had most suitable alleviation effect on crop yield under waterlogging stress.

Here, we implemented the first comprehensive global meta-analysis to evaluate the overall impacts of farmland management regimes (e.g. fertilization and growth regulators) on crop yields and biomass under waterlogging. The objectives of this study were to: (1) identify which management regimes have the most significant alleviating effect on crop yields under waterlogging stress; (2) determine which management regimes have the best alleviating effect on crop yields when waterlogging occurs at different growth stages; (3) assess the responses of varied crop yields to management regimes under waterlogging stress. This study aimed to offer a theoretical basis and technical support for the adoption of appropriate cultivation techniques in the events of waterlogging stress.

## 2 Materials and methods

### 2.1 Data collection

**Figure 1:**
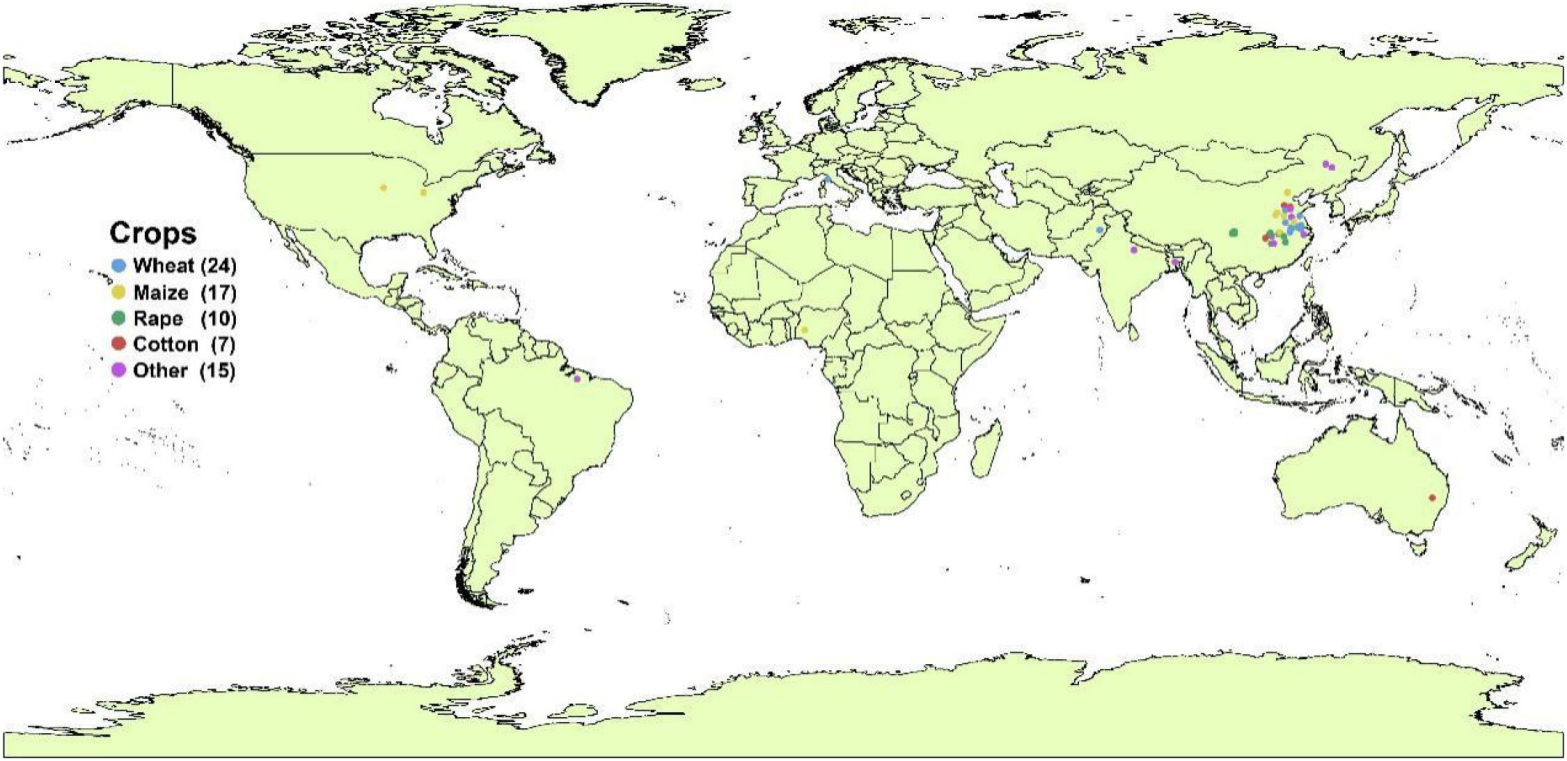
Locations of the experimental sites in the database.

A systematic exploration of the existing literature was performed via the China National Knowledge Infrastructure, Web of Science, and Google Scholar spanning the years 1980 to 2025. The search utilized “yield or biomass” in combination with “waterlogging or waterlogged or flooding” and “fertilizer or exogenous” as keywords to retrieve pertinent studies. To meet the data requirements for conducting a meta-analysis, the following selection criteria are defined: (i) The experimental design should include waterlogging/flooding treatment and fertilizer or exogenous regulators, excluding well-watered condition; (ii) the research object in the published paper should be field crops (such as cereals, oilseeds, and fiber crops), horticultural and vegetables crops were excluded from the database thus to maintain agronomic consistency and comparability across studies.; (iii) the experimental conditions should be completed in a field or potted environment (**Supplementary Figure S1**). According to the aforementioned selection criteria, a total of 71 peer-reviewed publications (39 in Chinese and 32 in English) were selected for inclusion. In the global meta-analysis database, 338 data sets related to crop yield, 204 for thousand-grain weight, 263 for aboveground biomass, and 180 for root biomass were gathered. We observed that the data in the meta-analysis originated from Asia, Europe, Africa, South America, North America, and Oceania (**Supplementary Table 1**). Among them, the experiments established in Asia are mainly concentrated in the eastern region of China (**Figure 1**), mainly because the temporal and spatial distribution of rainfall in this region is uneven, with frequent and repeated occurrences of concentrated rainfall. The main field crops studied in the database were wheat and maize, indicating that these two crops are highly susceptible to waterlogging damage (Tian et al., 2021a). The data regarding crop yield and agronomic traits were either obtained from tables or extracted from figures with the aid of WebPlotDigitizer (Rohatgi, 2012). Additionally, other relevant information, such as the site location, soil fertility, and mean annual precipitation was also documented. To elucidate the impacts of other co-varying factors on yield and its components and agronomic traits, several key categorical factors were classified into the following groups to streamline the analysis (**Table 1**). Given the scarcity of research papers on other crops, bean, peanut, soybean, and sugarcane were combined into the “Others” category.

**Table 1:** Categories used in describing the environmental and management conditions.

Factor	Categories
Experimental condition	Field; Pot
Crop type	Wheat; Maize; Rape; Cotton; Others
Stage	Vegetative stage (VS); Reproductive stage (RS)
Management regime	Fertilizer; Exogenous regulator

Regarding crop yield and agronomic traits, the mean value (M), standard deviations (SD), and sample replications (N) of fertilizer/regulator treatment under waterlogging treatment were retrieved. In cases where only the standard errors (SE) were provided, the SD was computed using the following formula: SD=SE×√N. When both SD and SE were absent, the average coefficient of variation was determined based on the known mean and standard deviation across all data sets. Subsequently, the missing standard deviation within the data sets was filled (Zhao et al., 2016).

### 2.2 Meta-analysis

A random effects model was utilized to evaluate the influence of fertilizer/regulator regimes on crop yield and biomass, taking the waterlogging treatment as the control. The natural logarithm (lnR) of the response ratio was presented as the effect size (ES) for this meta-analysis (Hedges et al., 1999), which was computed by $\text{lnR}=\text{ln}(X_{t}/X_{c})$.

In this context, X_t_ and X_c_ denoted the response variable measurements under waterlogging for the treatment (fertilizer/regulator) and control groups, respectively (Hedges et al., 1999). The overall weighted response ratio (ln RR) and its bias-corrected 95% confidence intervals (CIs) were computed for the complete dataset and for subgroups using OpenMEE software (Wallace et al., 2017). A fertilizer/regulator response was considered statistically significant if the 95% bootstrap CI did not include zero. To facilitate interpretation, the ES was expressed as a percentage change, defined as follows:

$\text{Effect size}={\text{e}}^{\text{lnR}}-1$The relationship between yield change and nitrogen amount was quantified using regression analysis implemented with the lm package in R. We assessed publication bias by calculating fail-safe numbers. An N value greater than 5n + 10 (where n is the number of datasets) indicated a result robust to publication bias (Xiang et al., 2017).

## 3 Results

### 3.1 Effects of management regimes on crop yield and biomass under waterlogging stress

**Figure 2:**
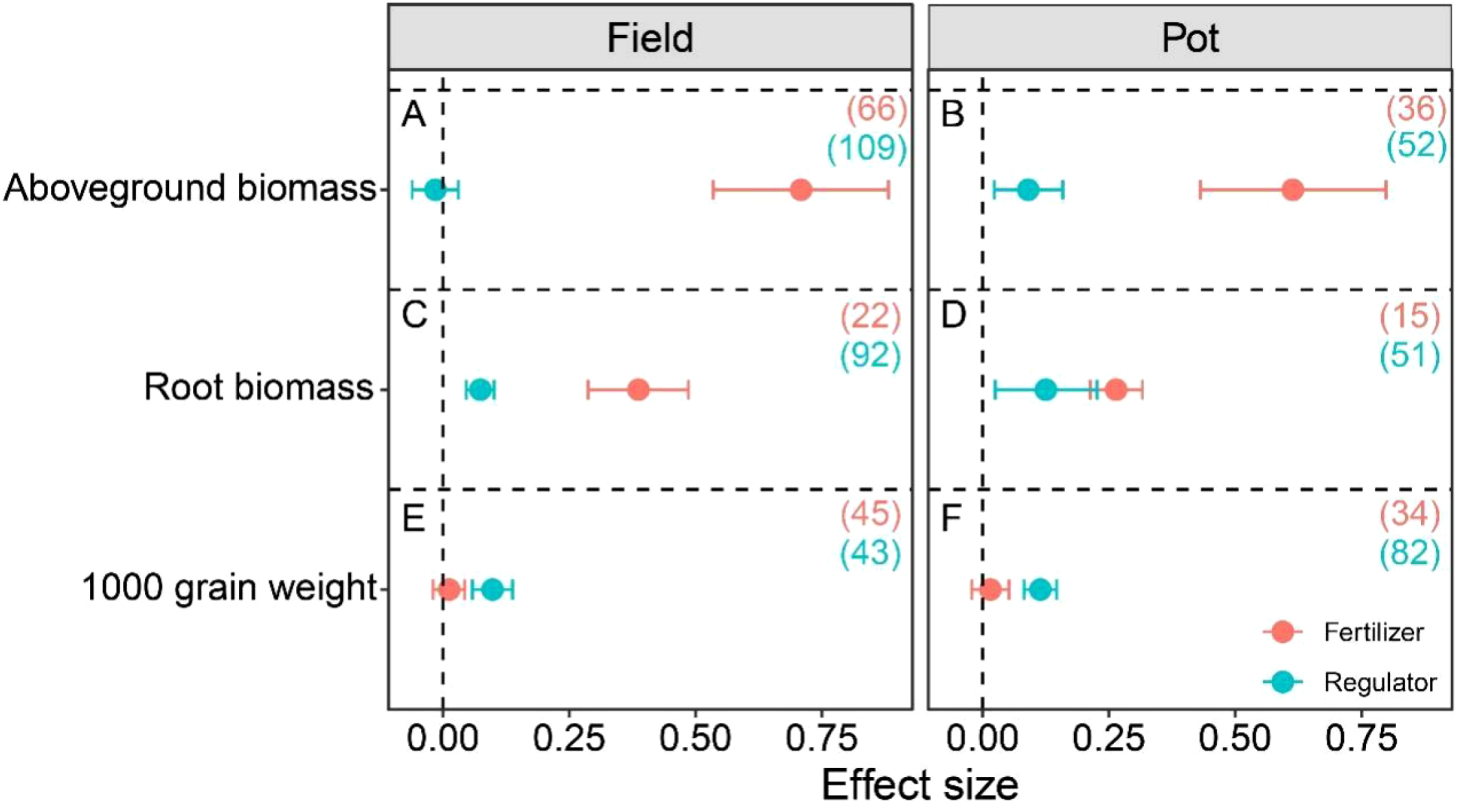
Effect size of management practices on aboveground biomass **(A, B)**, root biomass **(C, D)**, and 1000-grain weight **(E, F)** under waterlogging. The number of observations is displayed in parentheses. The horizontal bar indicates the 95% confidence interval (CI). An error bar that does not overlap 0 indicates a significant increase at *P <* 0.05.

Compared with waterlogging treatment, fertilizer treatment significantly increased the aboveground and root biomass under waterlogging (**Figures 2A–D**, p < 0.05), and under field and potted conditions, fertilizer treatment significantly increased aboveground biomass by 174.08% and 123.61%, respectively, and root biomass by 50.66% and 29.78%, respectively. Meanwhile, fertilizer treatment had a higher promoting effect on aboveground and root biomass than regulator treatment. Exogenous application of growth regulators significantly increased root biomass (**Figures 2C, D**) but had no effect on aboveground biomass under field conditions (**Figure 2A**).

**Figure 3:**
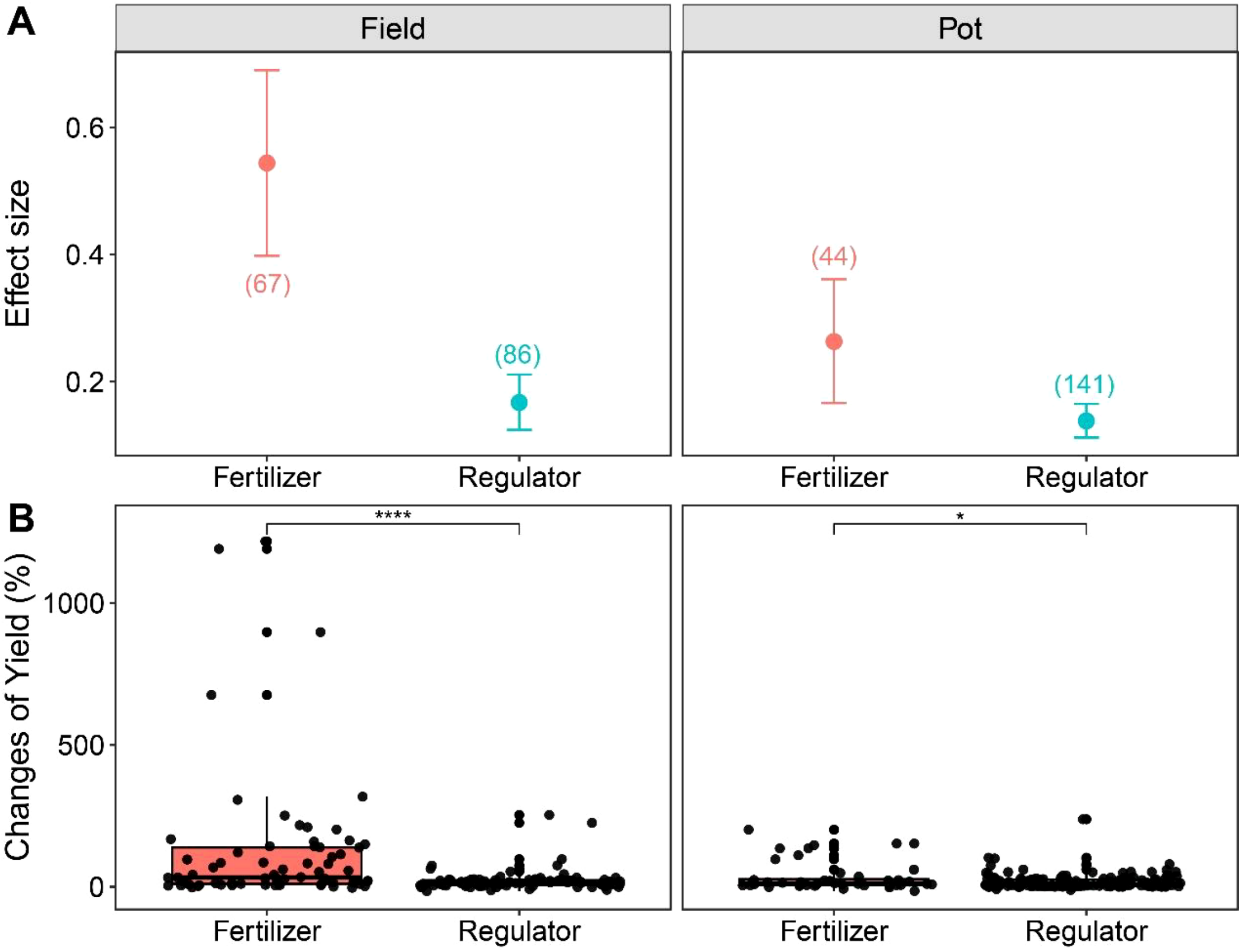
Effect size **(A)** and yield changes **(B)** of management practices on crop yields under waterlogging. The number of observations is displayed in parentheses. The vertical bar indicates the 95% confidence interval (CI). An error bar that does not overlap 0 indicates a significant increase at *P <* 0.05. The significance test between treatments is conducted using Wilcoxon test.

**Figure 4:**
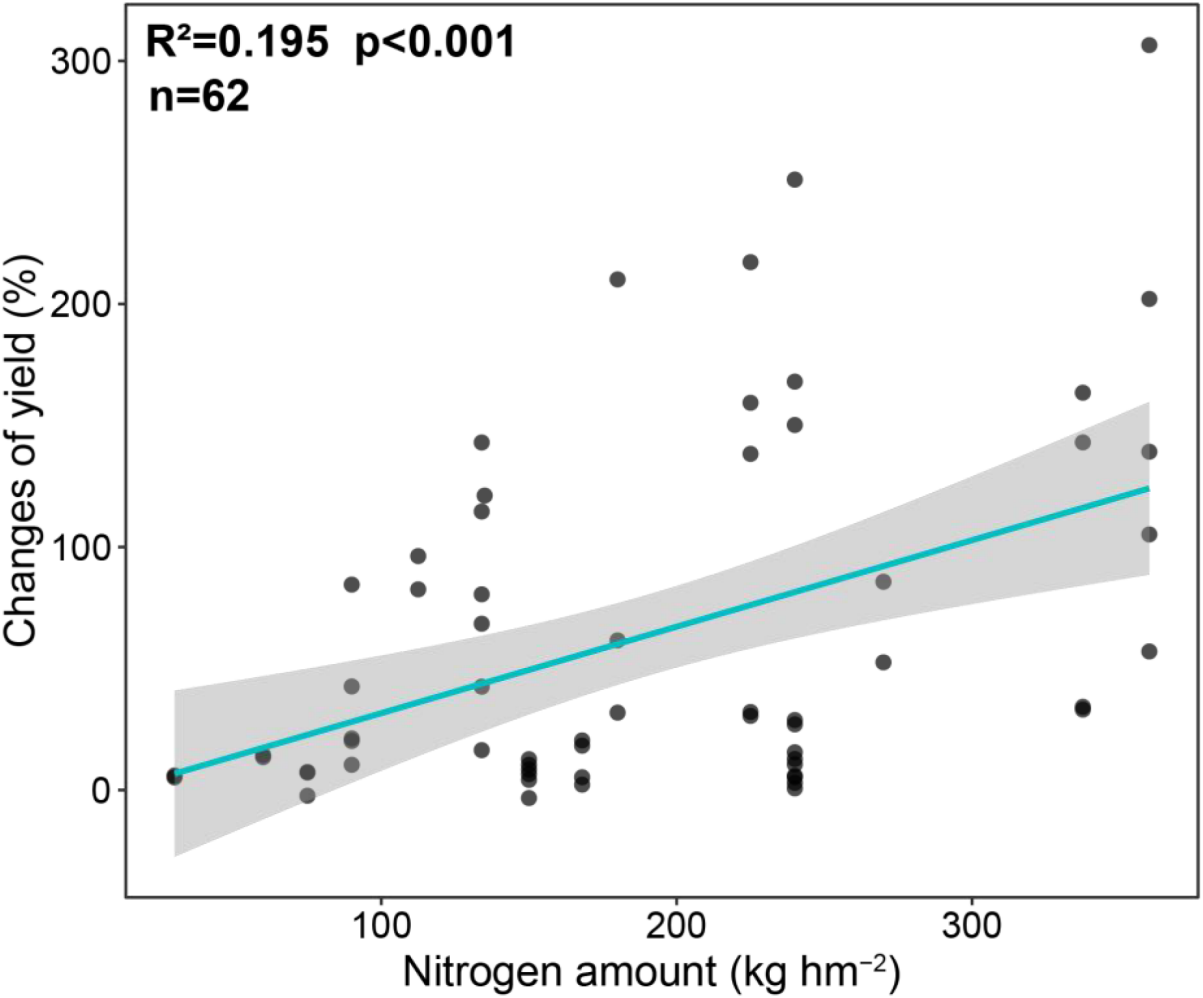
The relationship between changes of yield and nitrogen amount.

Under field and potted conditions, exogenous application of growth regulators significantly increased crop thousand grain weight by 11.05% and 14.90%, respectively, compared to waterlogging treatment alone (p < 0.05). However, fertilization treatment had no effect on thousand grain weight (**Figures 2E, F**). In terms of crop yield, both fertilizer and regulator treatment significantly increased crop yield compared to waterlogging treatment, and regardless of field or potted conditions, the crop yield increase of fertilizer treatment was greater than that of exogenous growth regulators (**Figure 3A**). Under field conditions, the average yield changes of fertilizer and regulator treatments were 123.32% and 21.93%, respectively, and under potted conditions, the average yield changes of fertilizer and regulator treatments were 36.20% and 17.88%, respectively (**Figure 3B**). Meanwhile, our results confirmed that with the increase of nitrogen fertilizer application, the variation of crop yield showed an increasing trend (**Figure 4**), although the relationship was significant, nitrogen amount explained 19.5% of the yield variation.

We also analyzed the use ways of growth regulators, and the results showed that whether in field or potted conditions, the application of regulators before or after waterlogging can significantly increase crop yield (**Supplementary Figure S2A**). Specifically, under field conditions, exogenous application of growth regulators before waterlogging can increase yield by 32.64%, while application of growth regulators after the relief of waterlogging stress can increase yield by an average of 17.03%; However, under potted conditions, the opposite trend was observed, with an average increase of 25.51% and 13.28% in yield after exogenous application of growth regulators before and after waterlogging stress (**Supplementary Figure S2B**). At the same time, in order to distinguish the regulatory effects of different growth regulator types on crop yield, we selected the top four growth regulators selected most in the database. The results showed that under waterlogging stress, the crop yield increased by 20.55%, 17.35%, 9.57%, and 9.34% under exogenous application of GA, 6-BA, S3307, and ABA, respectively (**Supplementary Figure S3**).

### 3.2 Effects of management regimes on crop yield at different growth stages under waterlogging stress

**Figure 5:**
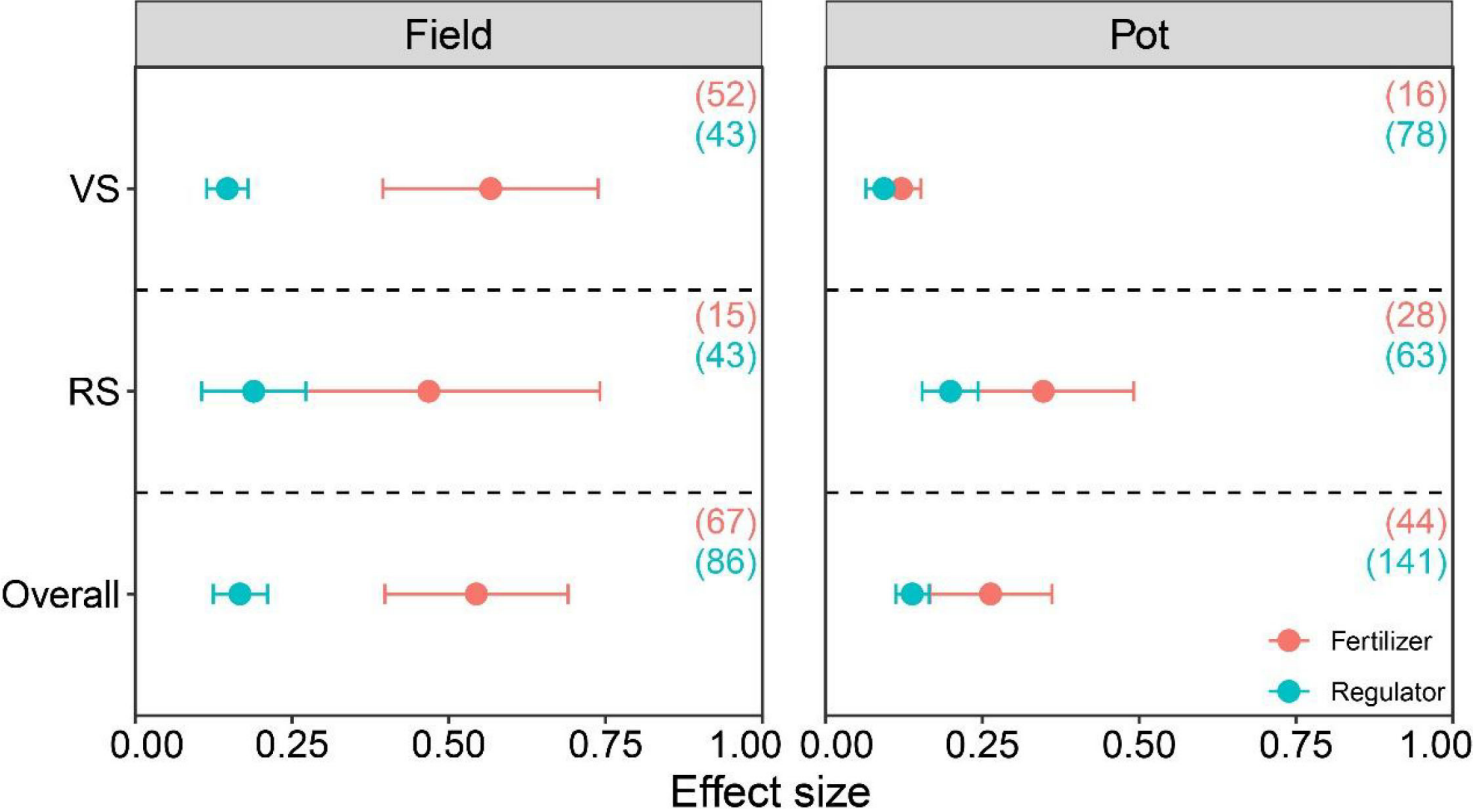
Crop yield response to management practices under waterlogging in different growth stages. The number of observations is displayed in parentheses. VS, vegetative stage; RS, reproductive stage. The horizontal bar indicates the 95% confidence interval (CI). An error bar that does not overlap 0 indicates a significant increase at *P <* 0.05.

Regardless of whether waterlogging occurred during the vegetative or reproductive growth stages, fertilizer and regulator treatments can significantly increase crop yield (p < 0.05), and the use of fertilizers always led to a higher increase in yield than the regulators addition (p < 0.05). Specifically, under field conditions, waterlogging treatment during the VS period, the addition of fertilizer and regulator increased the crop yield by 134.50% and 16.55%, respectively; During the RS period of waterlogging treatment, the use of fertilizers and regulators added the crop yield by 84.57% and 27.31%, respectively (**Figure 5**). Under potted conditions, the effects of fertilizer and regulator on crop yield increase during the VS period of waterlogging are similar.

### 3.3 Effects of management regimes on crop yield among varied crop types under waterlogging stress

**Figure 6:**
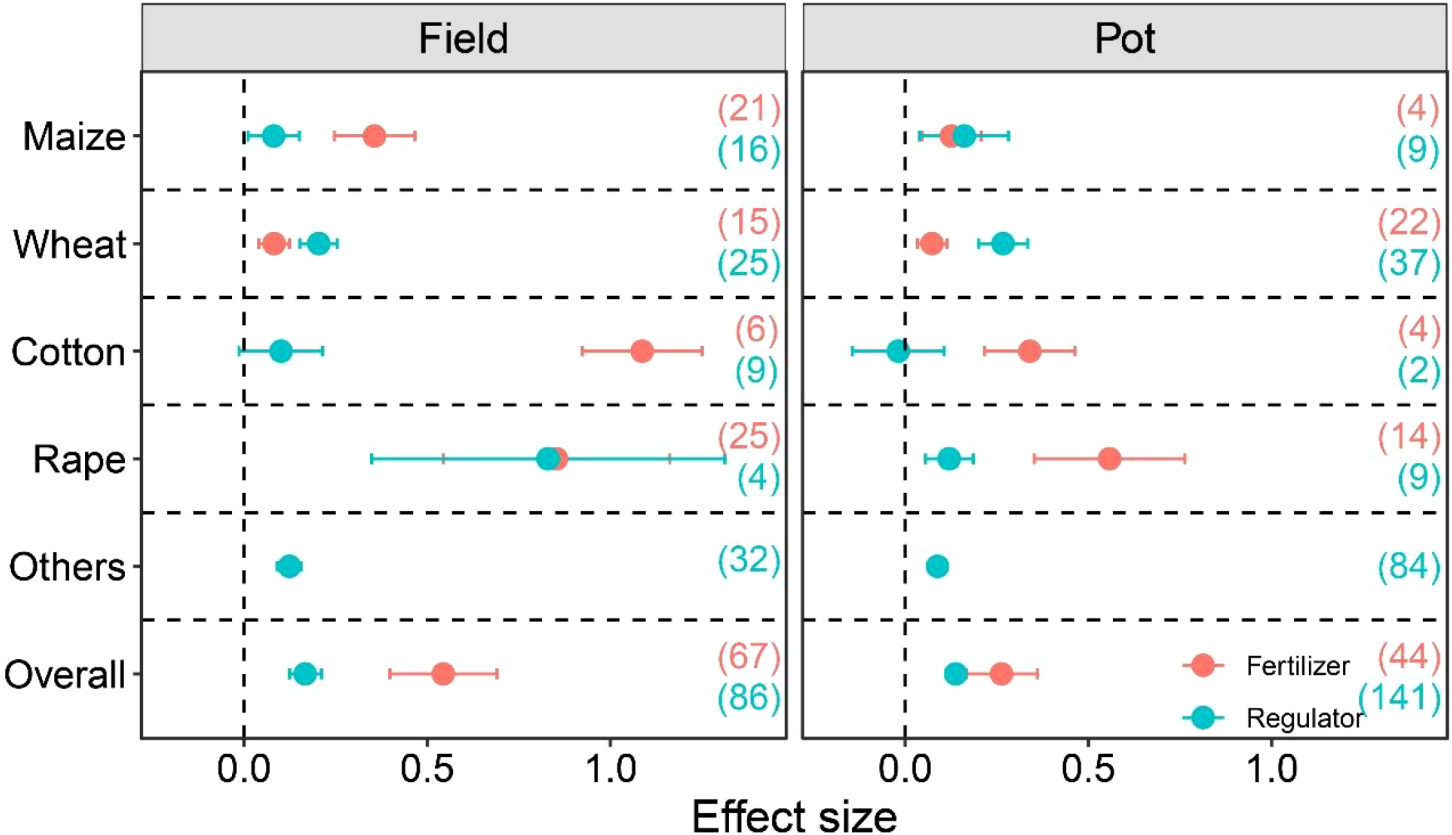
Crop yield response to management practices under waterlogging in different Crop types. The number of observations is displayed in parentheses. VS, vegetative stage; RS, reproductive stage. The horizontal bar indicates the 95% confidence interval (CI). An error bar that does not overlap 0 indicates a significant increase at *P <* 0.05.

In both field and pot trials, the influence of fertilizer and regulator application on crop yield was notably affected among the distinct crop types (**Figure 6**). We observed that under waterlogging stress, fertilizer treatment significantly increased cotton yield, but the effect of exogenous growth regulators on cotton yield was not significant. Meanwhile, among other crops, only growth regulators have been studied for their yield. Regardless of field or potted conditions, the application of exogenous growth regulators always resulted in a higher increase in wheat yield than fertilizer treatment, and the application of exogenous regulators significantly increased wheat yield by 23.51% and 36.07%, respectively, while fertilizer treatment increased wheat yield by 8.83% and 7.12%, respectively. For maize, more researches were focused on field studies, and fertilizer treatment has a higher increase in maize yield (47.49%) than that of regulator treatment (9.48%).

## 4 Discussion

### 4.1 Mechanisms underlying the superiority of fertilization

This meta-analysis integrated 338 data sets of crop yields from 71 papers, and the results showed that both fertilization and exogenous growth regulators can effectively alleviate crop yield reduction caused by waterlogging stress, but the overall effect of fertilization was significantly better than that of growth regulators, indicating fertilization can effectively mitigate the impact of waterlogging stress on crop yield and narrow the yield gap between waterlogged conditions and normal water management practices (Hu et al., 2023). This is mainly attributed to the following aspects: Firstly, fertilization showed a strong promoting effect on the accumulation of crop biomass. Under field conditions, fertilization significantly increased aboveground biomass and root biomass by 174.08% and 50.66%, respectively (**Figures 2A–D**), indicating fertilization enhanced the overall growth potential and assimilation ability of crops, laying a solid material foundation for the final yield formation (Xing et al., 2025). In contrast, the effect of exogenous growth regulators on increasing aboveground biomass is not significant under field conditions, although they have a certain promoting effect on root biomass. Secondly, regardless of field or potted conditions, the increase in yield brought by fertilization is much higher than that of growth regulators treatment (**Figures 3A, B**). This difference may be due to the vastly different mechanisms of action between the two: fertilization mainly supplemented essential nutrients (especially nitrogen) that are lost in soil or obstructed by crop absorption under waterlogging stress, directly alleviating nutritional stress, maintaining normal physiological metabolism and growth and development (Zhao et al., 2025). Our regression analysis further confirmed that the increase in crop yield was enhanced with the increase of nitrogen fertilizer application (**Figure 4**), which is consistent with the research results of Tian et al. (2021b), that nitrogen fertilizer can regulate the photosynthetic characteristics of maize leaves, enhance nitrogen use efficiency, alleviate the inhibition of yield by waterlogging, and highlight the core position of nutrient supply in stress resistance. Growth regulators mainly improved the physiological state of crops by regulating endogenous hormone levels (Ahmad et al., 2025), reducing oxidative damage (Jiang et al., 2025) or regulating stomatal behavior (Qiu et al., 2024), and their effects may be more indirect and limited. Although growth regulators have shown unique advantages in increasing thousand grain weight (**Figures 2E, F**), their driving force for overall biomass accumulation is insufficient, resulting in a lower contribution to final yield compared to fertilizer regime. Fertilization treatment can stably exert its effect under field and potted experimental conditions, while the effect of growth regulators is greatly affected by the timing of application: under field conditions, the increase in application before waterlogging (32.64%) is higher than that after waterlogging (17.03%), while under pot conditions, the opposite trend is shown (25.51% before waterlogging and 13.28% after waterlogging), this difference in yield results may be attributed to the difference in the severity of waterlogging stress under field and pot conditions, and the limited space for crop root growth under pot conditions. Meanwhile, this instability also further highlighted the advantages of fertilization in alleviating waterlogging. In general, fertilization achieves efficient alleviation of waterlogging stress through a complete regulatory chain of “nutrient supply-biomass accumulation-yield formation”. Therefore, in practices aimed at quickly restoring crop growth and minimizing yield losses, optimizing fertilization strategies should be considered as a more direct and effective primary management practices than applying exogenous regulators.

### 4.2 Differential response of crop yield to management practices during different waterlogging periods

The timing of waterlogging stress significantly influenced the responses of crop yields to fertilization and growth regulators, and this difference is closely related to the physiological needs and metabolic characteristics of crops at different growth stages (Huang et al., 2024). Our findings demonstrated that regardless of whether waterlogging occurred during the vegetative stage or the reproductive stage, the yield increase induced by fertilization is consistently greater than that from growth regulators. Specifically, when waterlogging occurred during the vegetative stage, fertilization under field conditions resulted in a substantial yield increase of 134.50%, far exceeding the 16.55% increase achieved with growth regulators (**Figure 5**). The vegetative stage is a critical period for crops to establish their photosynthetic system and root architecture; waterlogging during this phase primarily severely hampered the development of roots, stems, and leaves (Daniel and Hartman, 2024). Fertilization likely facilitated rapid recovery and compensatory growth after the alleviation of waterlogging stress by timely nutrient supply, leading to significant yield gains. When waterlogging occurred during the reproductive stage, the yield increase from fertilization (84.57%), although lower than during the vegetative stage, still significantly surpassed that from growth regulators (27.31%). The reproductive stage is crucial for determining yield components such as number of spikes, number of grains, and grain weight, and is extremely sensitive to stress. At this time, waterlogging is more likely to affect pollen vitality, fertilization process, and grain filling efficiency (Zhang et al., 2025). Fertilization may ensure the supply of photosynthetic products and their transport to grains. However, the yield increase is constrained due to the irreversible nature of damage to reproductive organ development, such as pollen abortion caused by waterlogging at flowering, which is difficult to reverse (Yang et al., 2023). It is noteworthy that the relative effectiveness of grow regulators improved during the reproductive stage compared to the vegetative stage. This may be because crops are more sensitive to hormonal regulation during this period, and the application of growth regulators can be applied to delay leaf senescence or regulate the distribution of photosynthetic products, thereby more directly affecting the yield formation process (Huang et al., 2024). Furthermore, under potted conditions, the difference in yield increase between fertilization and growth regulators is relatively small when waterlogging occurred during the vegetative stage, which may be related to the low nutrient loss and limited crop growth space in the potted environment, making it difficult to fully utilize the nutritional advantages of fertilization. In conclusion, fertilizer regime resulted in a greater absolute yield increase than regulators application at both stages, underscoring the fundamental role of nutrient management in mitigating waterlogging stress throughout the crop growth cycle. This finding provides crucial guidance for farmers to precisely select mitigation measures based on the timing of waterlogging occurrence.

### 4.3 Differential responses of crop yield of varied crop types to management practices under waterlogging conditions

The differences in response of different crop types to waterlogging mitigation measures are mainly due to the genetic characteristics, physiological metabolic mechanisms, and cultivation purposes of crop resistance to waterlogging. This study revealed significant differences in how different crops respond to fertilization and growth regulators applications, underscoring the necessity of tailoring measures to specific crops. Exogenous growth regulators brought stable wheat yield increases of 23.51% and 36.07% under field and potted conditions, respectively, which were better than the fertilizer regime (**Figure 6**). As a cereal crop, wheat’s yield is particularly sensitive to grain filling efficiency (Ullah et al., 2024). Growth regulators may effectively promote the grain filling process by optimizing endogenous hormone balance, resulting in an advantage in thousand grain weight (**Figures 2E, F**), ultimately leading to higher yields. Maize showed a strong dependence on fertilization, with a yield increase of 47.49% under field conditions being much higher than that of growth regulators (9.48%). Maize, as a C4 crop with high light utilization efficiency, requires extremely high nutrient requirements for biomass accumulation and yield formation (Sales et al., 2021). Waterlogging can severely inhibit the absorption of nitrogen, phosphorus, and other elements by its roots (Ahmad et al., 2025). Fertilization can directly supplement nutrient gaps, promote the development of maize female ears, and increase the number of grains per ear. However, growth regulators are difficult to meet their huge nutritional needs, so the effect is limited. The response pattern of cotton is even more unique: cotton showed a significant response to fertilization, while growth regulators has no significant effect on its yield. Cotton, as a dicotyledonous crop, has strict requirements for nitrogen and potassium in its fiber development (Ashfaq et al., 2015; Shah et al., 2022). Nutrient loss caused by waterlogging directly affected the development of cotton bolls; Fertilization can alleviate waterlogging damage by increasing the nutrient level of cotton plants, increasing the number of bolls and fiber length (Qi and Wu, 2023). Therefore, when formulating strategies for waterlogging reduction, it is necessary to fully consider the specificity of the target crops.

### 4.4 Limitations and future research

Although current research provides insights into understanding how management measures mitigated the negative impacts of waterlogging, data coverage is fraught with biases, such as an overwhelming preponderance of data from East Asia (especially China), while data from other regions are scarce, which severely limit the generalizability of the results to different global climates and soil conditions. Additionally, the research on different crop types is skewed, making it difficult to comprehensively capture the differences in how various crops respond to management measures. We propose conducting more in-depth research on the alleviation strategies for management practices amidst soil waterlogging in numerous regions and different types of crops to improve the applicability and robustness of future analysis. Additionally, the intrinsic physiological mechanism of the effectiveness of fertilization or exogenous growth regulators on yield loss under waterlogging needs to be fully explained, which highlights the necessity of future research exploring the impact of these two management measures on crop growth and yield, so as to provide more in-depth theoretical support for precise waterlogging-resistant management.

## 5 Conclusion

This study revealed that both fertilization and exogenous application of growth regulators can increase crop yield under waterlogging, but the effects of fertilization on alleviating yield loss is greater than that of growth regulators treatment, which is attributed to the significant promoting effect of fertilization on the accumulation of root and shoot biomass, while growth regulators have a comprehensive effect on increasing thousand grain weight and crop yield. Furthermore, the magnitude of the increase in crop yield induced by fertilizer regime increased with the increase in nitrogen amount. When waterlogging stress occurred during the reproductive growth period, the regulatory effect of fertilization on crop yield was superior to the exogenous application of growth regulators. We also observed that under waterlogging conditions, the effect of exogenous growth regulators on cotton yield was not significant. Moreover, the positive effect of exogenous growth regulators on increasing wheat yield is more significant than that of fertilization. In total, this meta-analysis deepens our understanding of management practices to alleviate yield losses caused by waterlogging, and highlights fertilizer regime for maintaining and increasing crop yields under challenging environmental conditions.

## Data Availability

The raw data supporting the conclusions of this article will be made available by the authors, without undue reservation.
