# Supplementary material for: Fertilization is more effective in alleviating yield loss of waterlogged crops than exogenous growth regulators: a meta-analysis
Source: Front Plant Sci. 2026 Mar 11;17:1779872. doi: 10.3389/fpls.2026.1779872 (PMC13012985; doi:10.3389/fpls.2026.1779872)
Supplement: Supplementary file 1 [file Table1.docx]

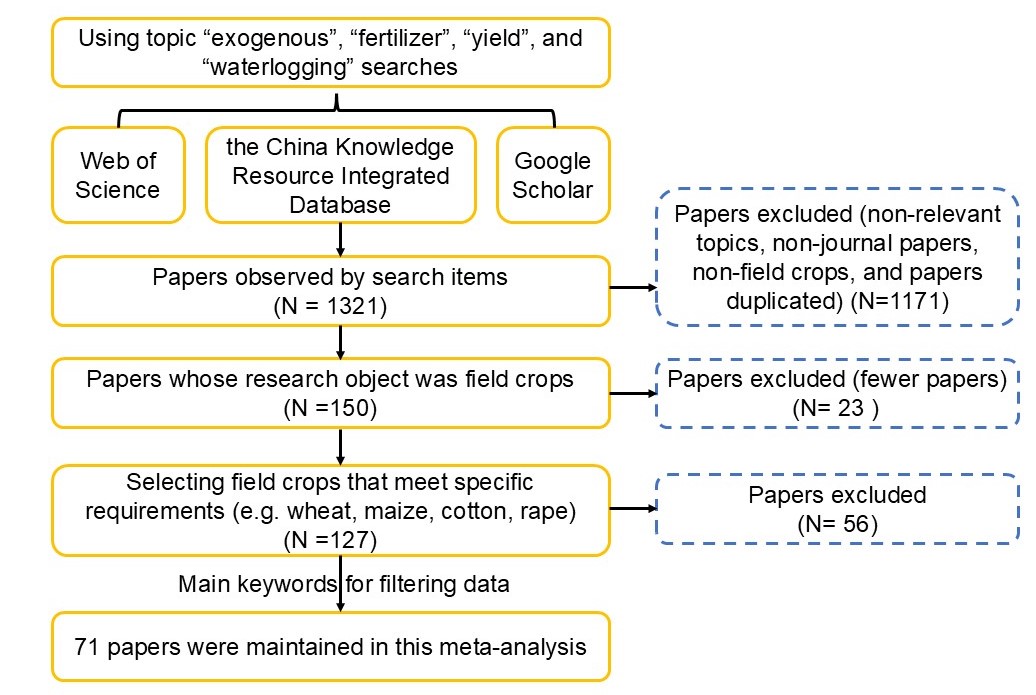


Figure S1 Approach invoked for identification, screening and selection of the datasets for meta-analysis in this study.


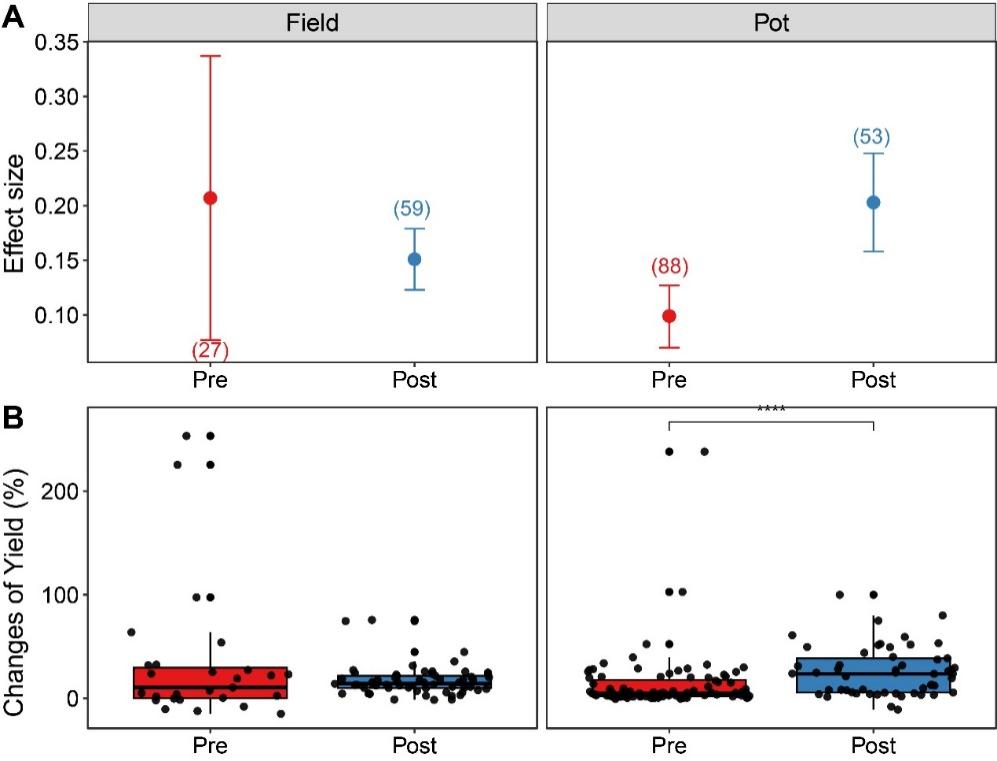


Figure S2 Effect size (A) and yield changes (B) of the application of growth regulators before or after waterlogging treatment on crop yields. Pre represents the application of regulators before waterlogging treatment; Post represents the application of regulators after waterlogging treatment. The number of observations is displayed in parentheses. The horizontal bar indicates the 95% confidence interval (CI). An error bar that does not overlap 0 indicates a significant increase at *P <* 0.05. The significance test between treatments is conducted using Wilcoxon test.


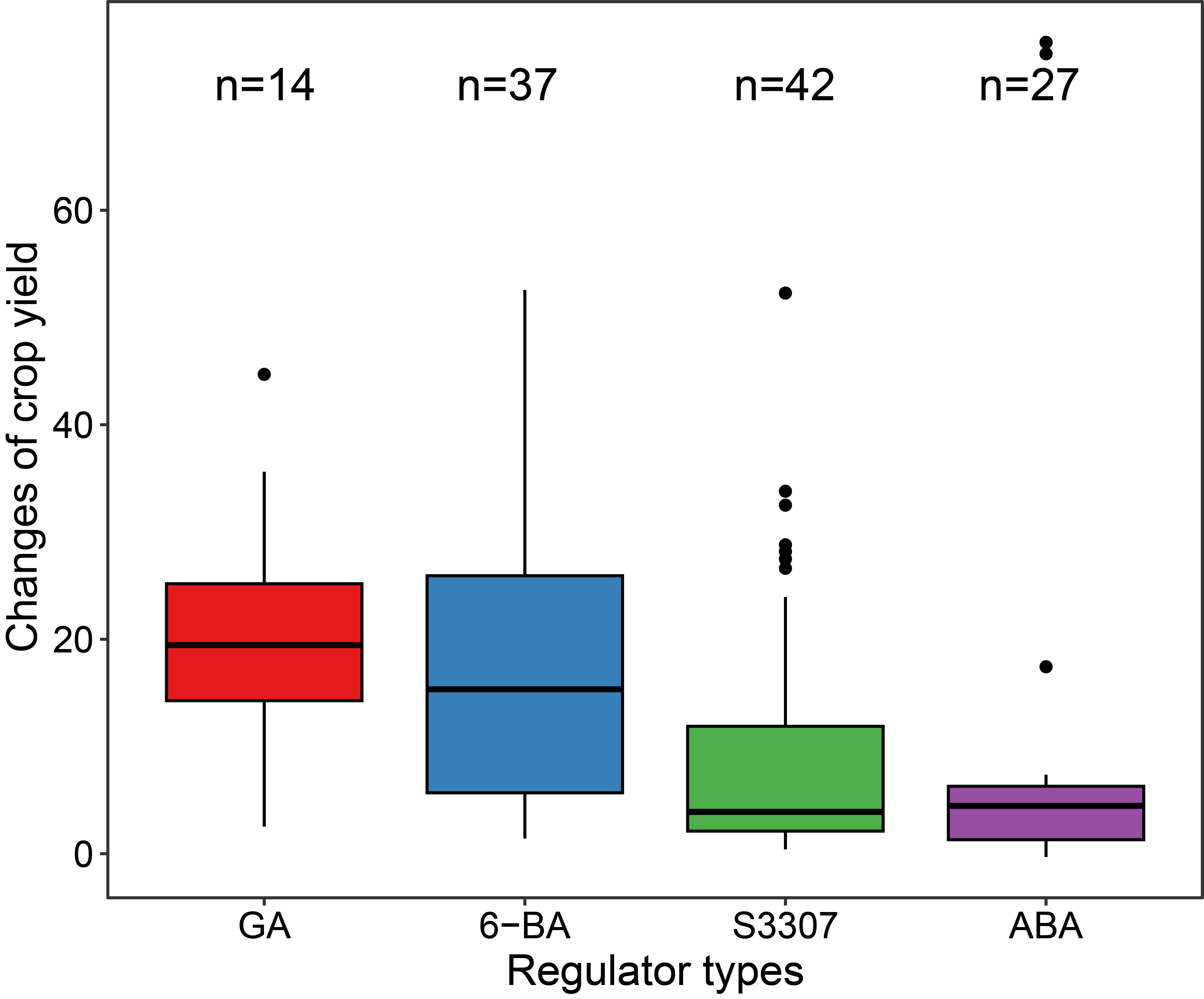


Fig. S3 Effects of different hormone types of growth regulators on crop yield under waterlogging stress

Table S1 Global distribution of data sets

| Continent | No. of data points | Percentage of total data points |
| --- | --- | --- |
| Asia | 943 | 95.73% |
| Europe | 4 | 0.41% |
| Africa | 1 | 0.10% |
| South America | 4 | 0.41% |
| North America | 22 | 2.23% |
| Oceania | 11 | 1.12% |

Table S2 Publication bias was tested using fail-safe numbers.

| Variables | Rosenthal's Fail-safe Number | | 5n+10 | Publication bias |
| --- | --- | --- | --- | --- |
|  | Number of observations (n) | Fail-safe number (N) |  |  |
| Yield | 338 | 3717803 | 1700 | No |
| 1000-grain weight | 204 | 1335317 | 1030 | No |
| Aboveground biomass | 263 | 606525 | 1325 | No |
| Root biomass | 180 | 123055 | 910 | No |

Fail-safe N analysis was not conducted for oil content study, because this effect size was not significant
